# Supplementary material for: 2-Phenylpyridine Derivatives: Synthesis and Insecticidal Activity against Mythimna separata, Aphis craccivora, and Tetranychus cinnabarinus
Source: Molecules. 2023 Feb 6;28(4):1567. doi: 10.3390/molecules28041567 (PMC9967329; doi:10.3390/molecules28041567)
Supplement: Supplementary file 1 [file molecules-28-01567-s001.zip › molecules-2146244-supplementary.pdf]

Article

Synthesis and insecticidal agents of 2-phenylpyridine compounds containing N-phenylbenzamide moieties

Wenliang Zhang, Jingjing Chen and Xiaohua Du

Catalytic Hydrogenation Research Center, Zhejiang Key Laboratory of Green Pesticides and Cleaner Production Technology, Zhejiang Green Pesticide Collaborative Innovation Center, Zhejiang University of Technology, Hangzhou 310014, P. R. China

* Correspondence: duxiaohua@zjut.edu.cn (X.-H.D.)

Supporting Information

Contents

1. ^{1}H NMR spectra and ^{13}C NMR spectra of target compounds 5a-5k 2

2. [1] H NMR spectra and ^{13}C NMR spectra of target compounds 5a-5k

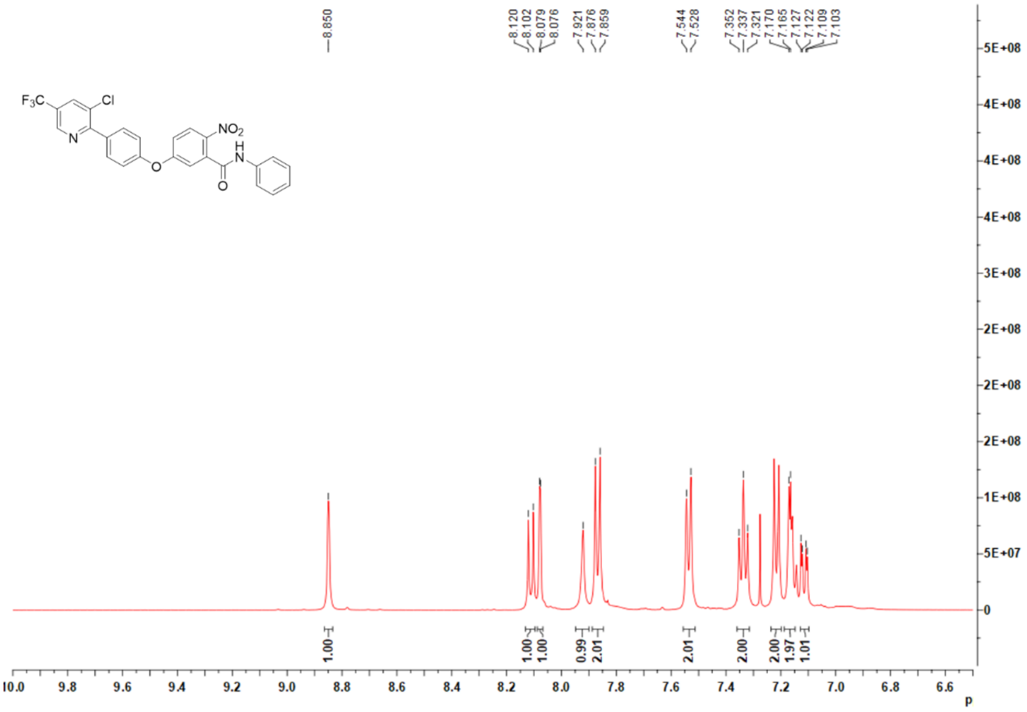

Figure S1. The ^{1}H NMR spectrum of **5a.**

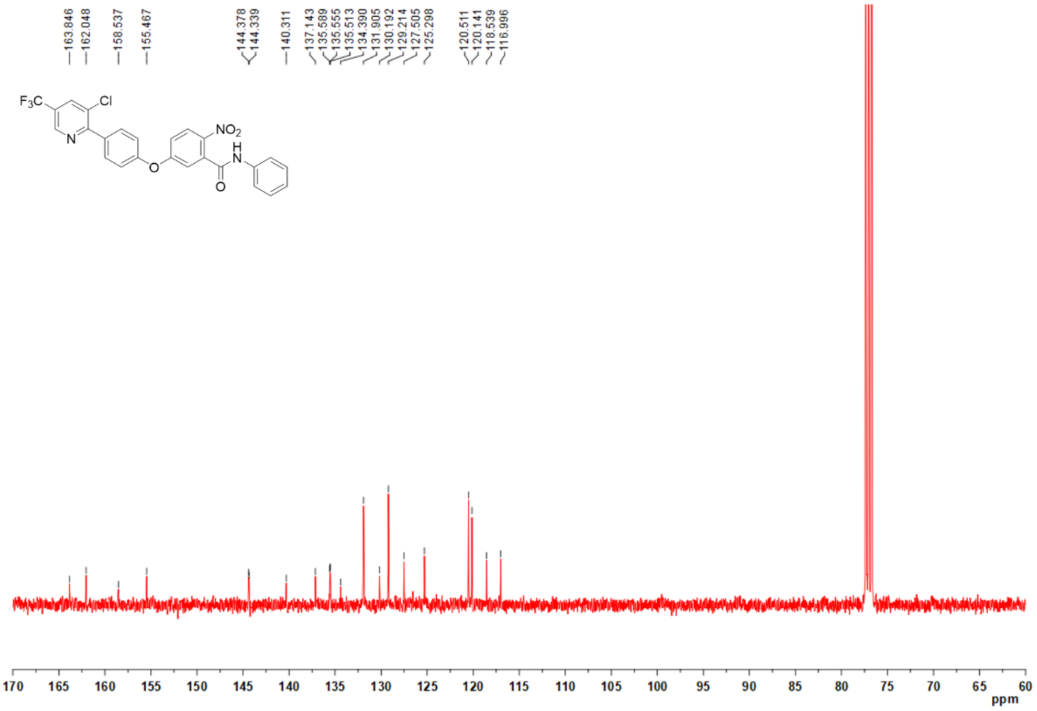

Figure S2. The ^{13}C NMR spectrum of **5a.**

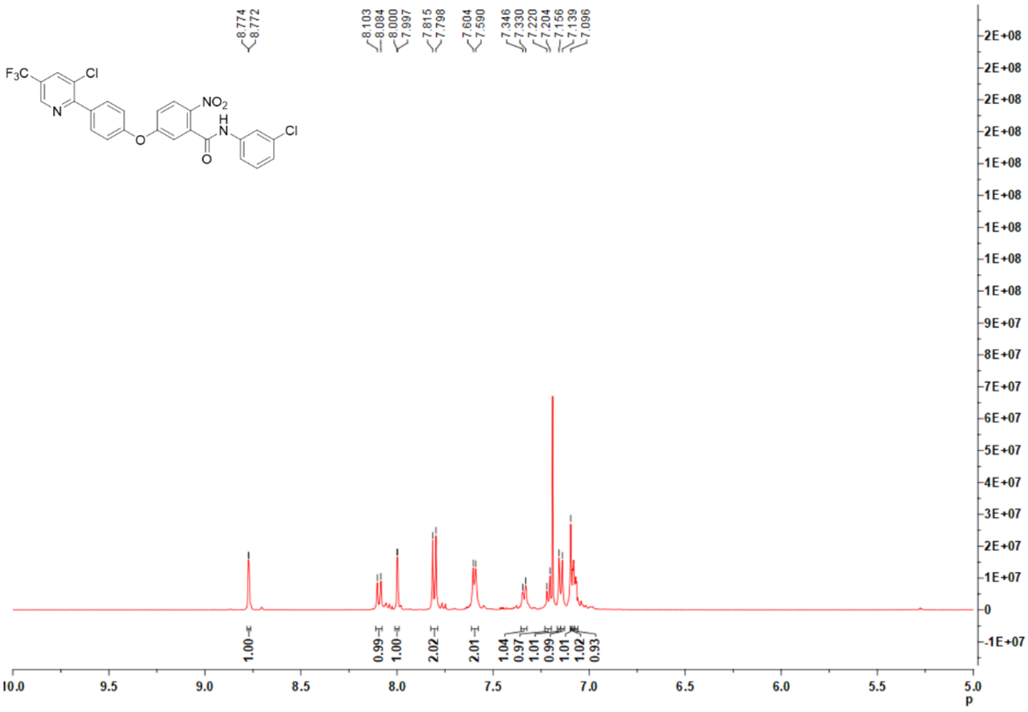

Figure S3. The ^{1}H NMR spectrum of **5b.**

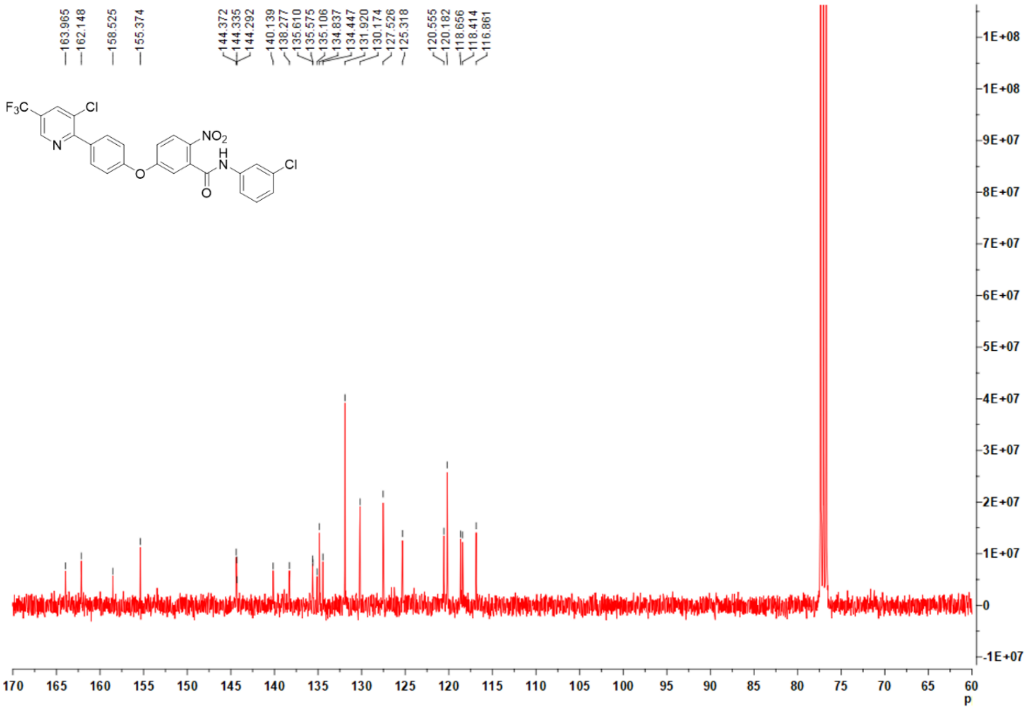

Figure S4. The ^{13}C NMR spectrum of **5b.**

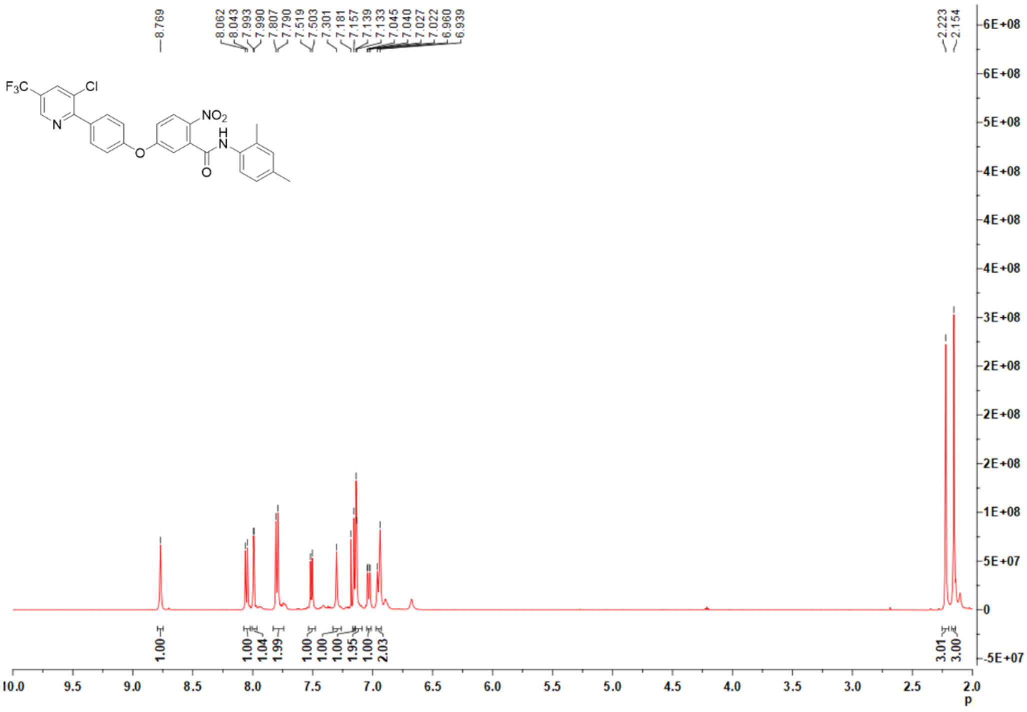

Figure S5. The ^{1}H NMR spectrum of **5c.**

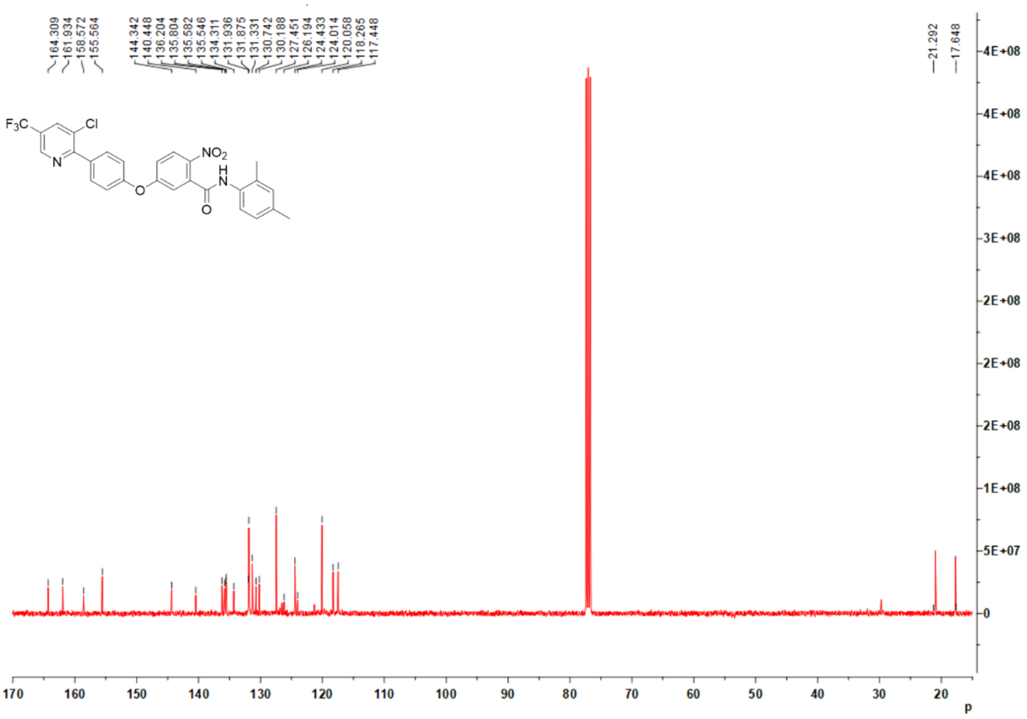

Figure S6. The ^{13}C NMR spectrum of **5c.**

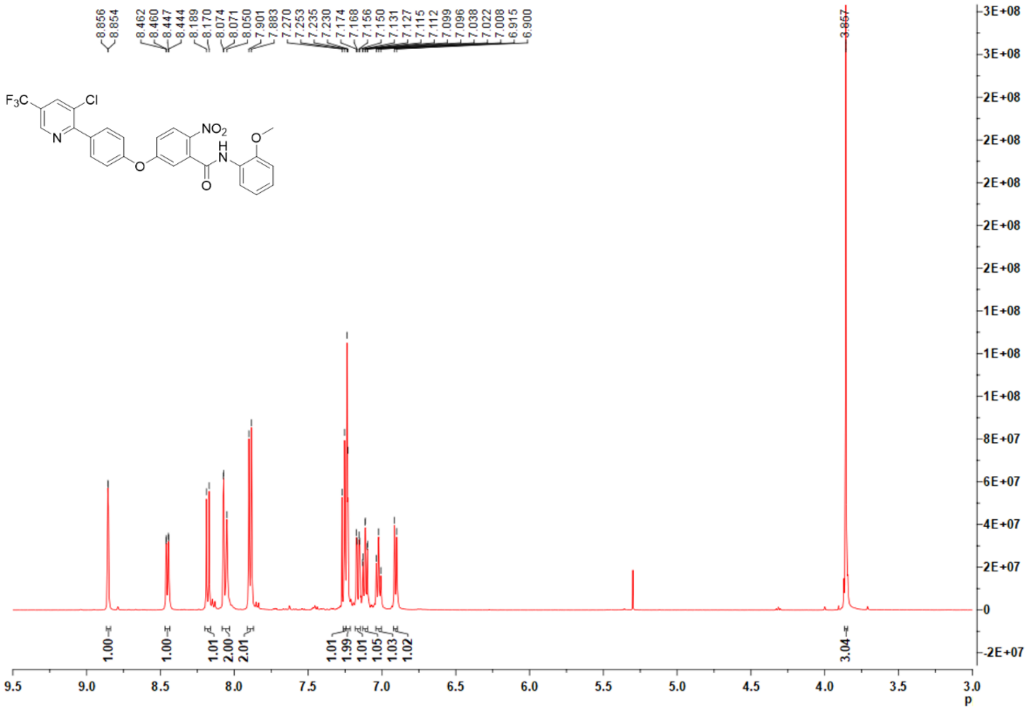

Figure S7. The ^{1}H NMR spectrum of **5d.**

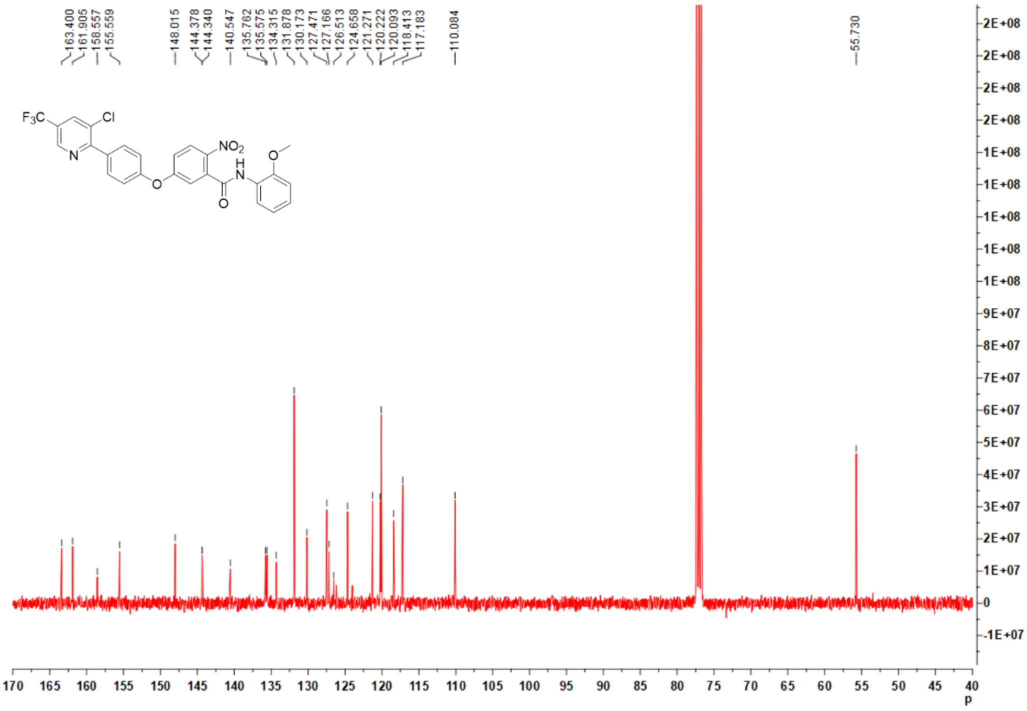

Figure S8. The ^{13}C NMR spectrum of **5d.**

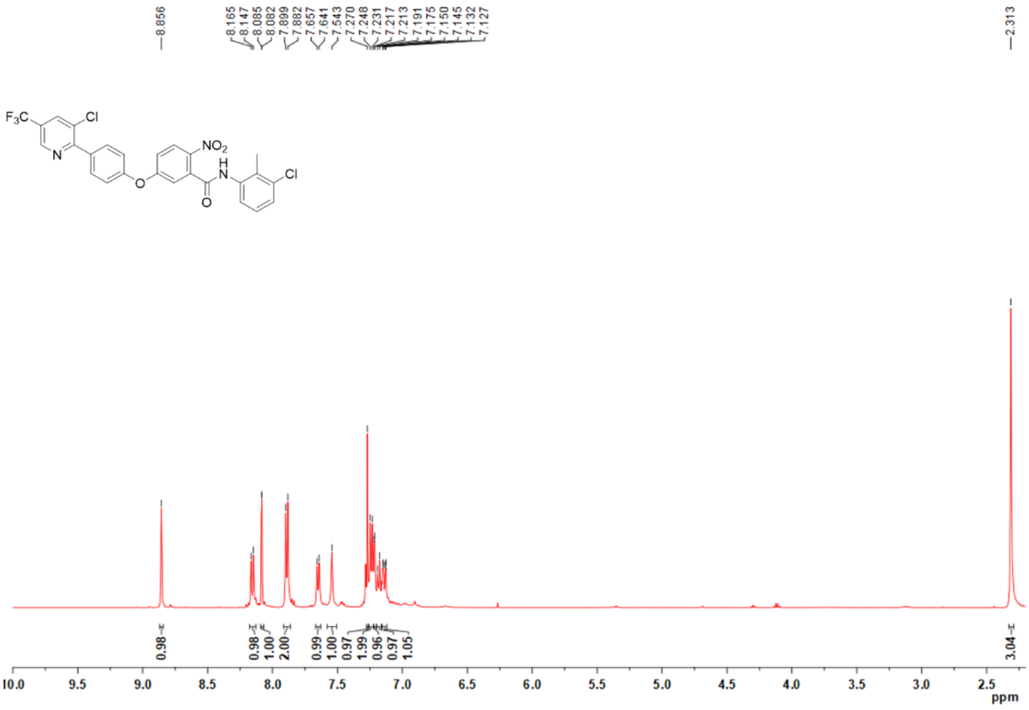

Figure S9. The ^{1}H NMR spectrum of **5e.**

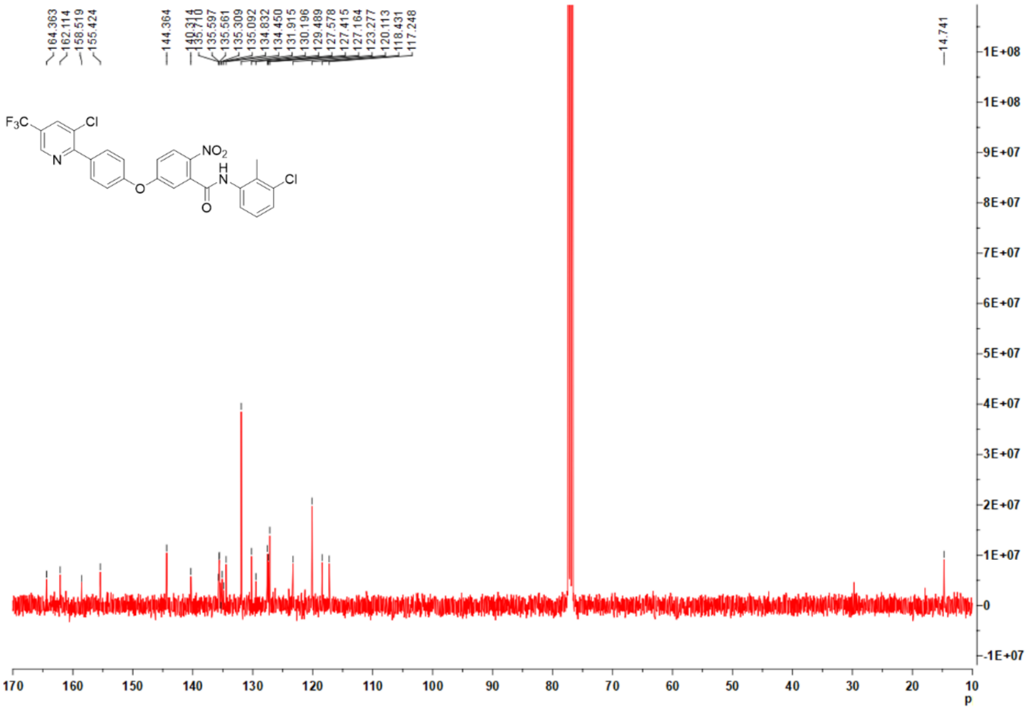

Figure S10. The ^{13}C NMR spectrum of **5e.**

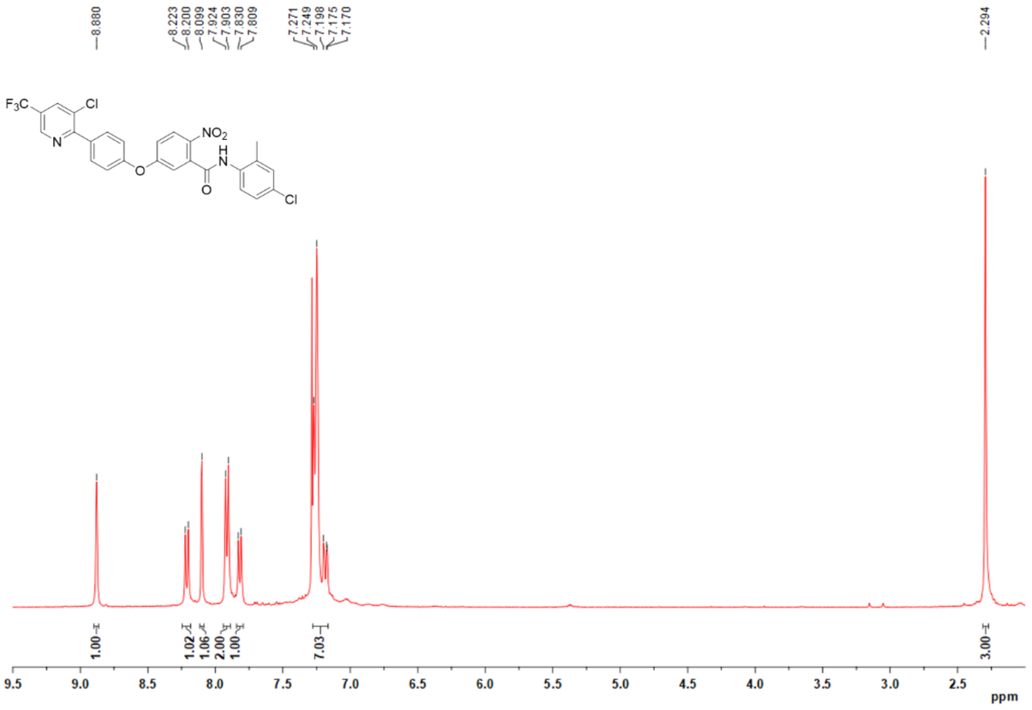

Figure S11. The ^{1}H NMR spectrum of **5f.**

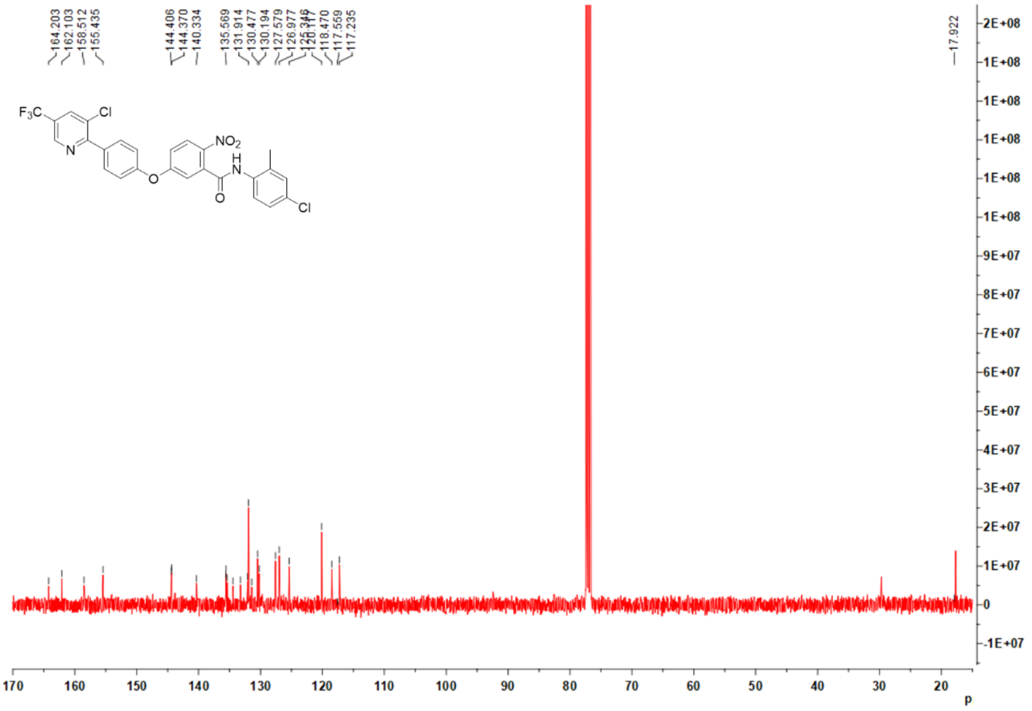

Figure S12. The ^{13}C NMR spectrum of **5f.**

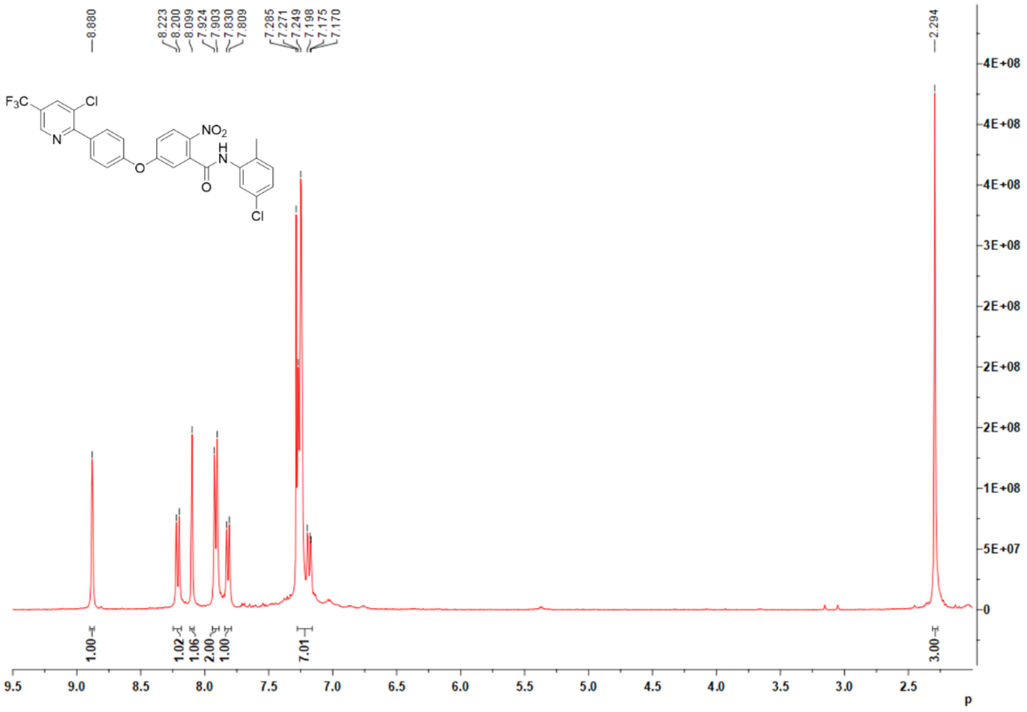

Figure S13. The ^{1}H NMR spectrum of **5g.**

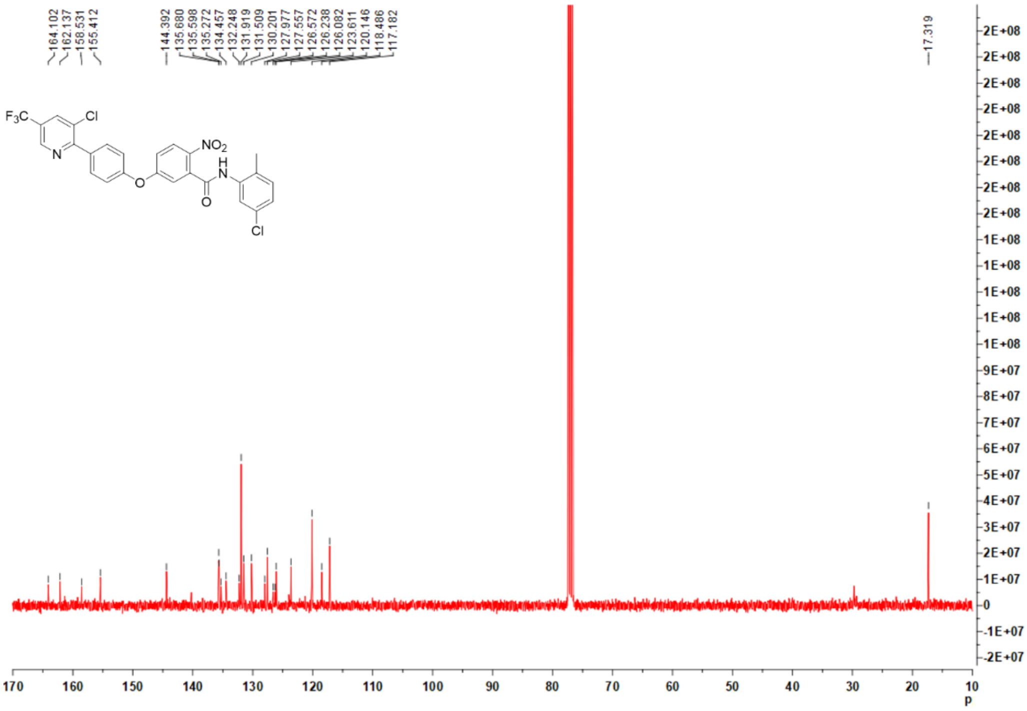

Figure S14. The ^{13}C NMR spectrum of **5g.**

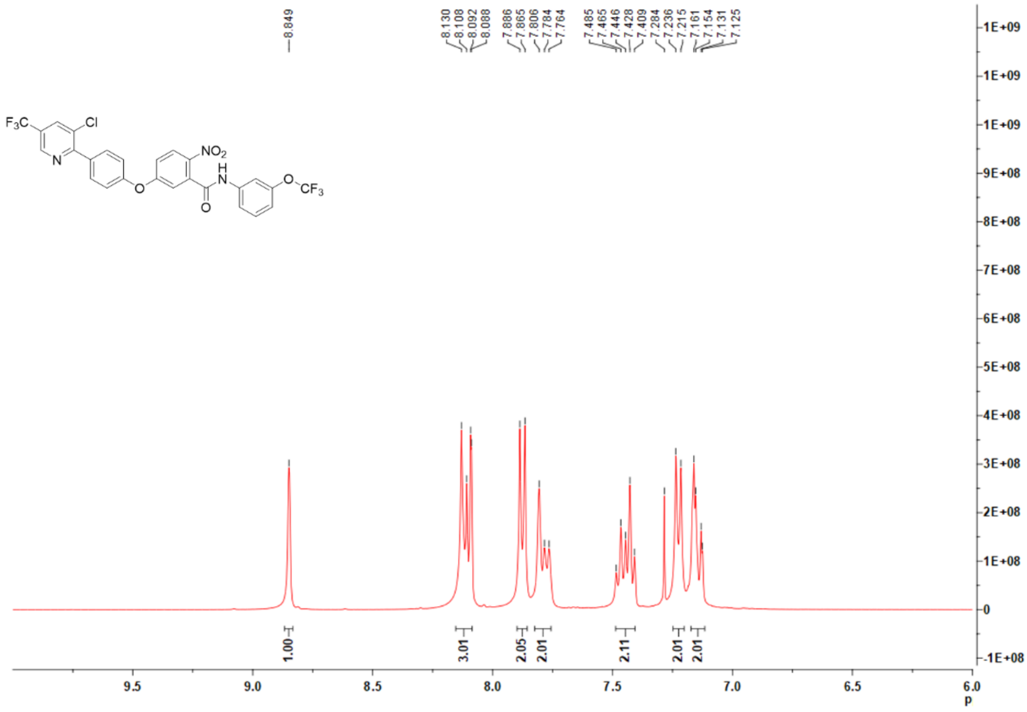

Figure S15. The ^{1}H NMR spectrum of **5h.**

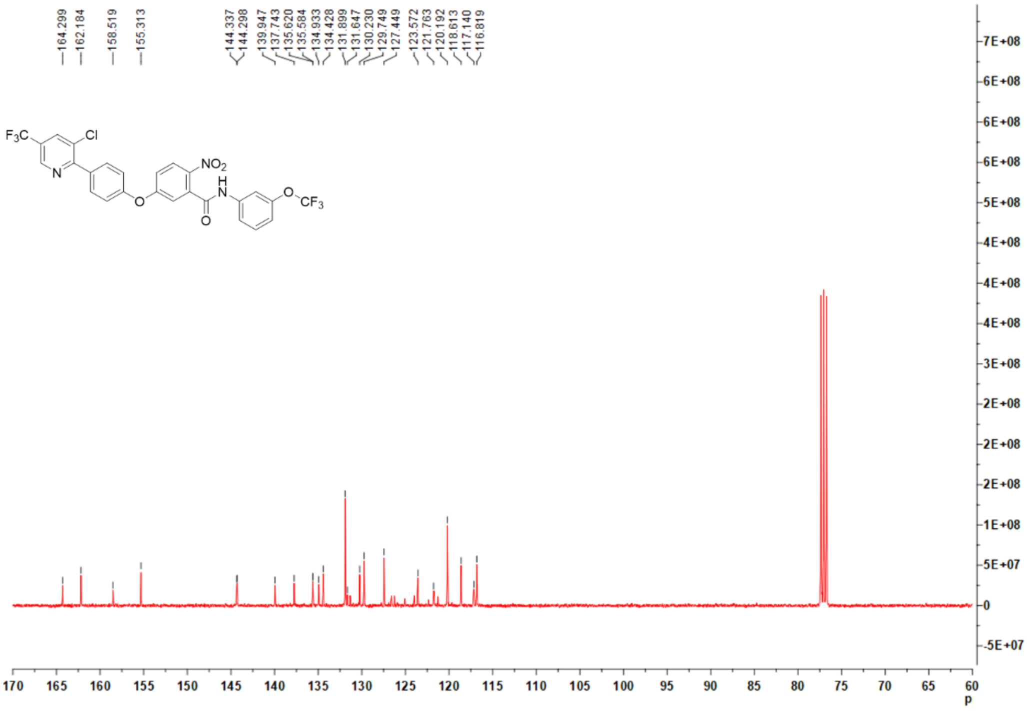

Figure S16. The ^{13}C NMR spectrum of **5h.**

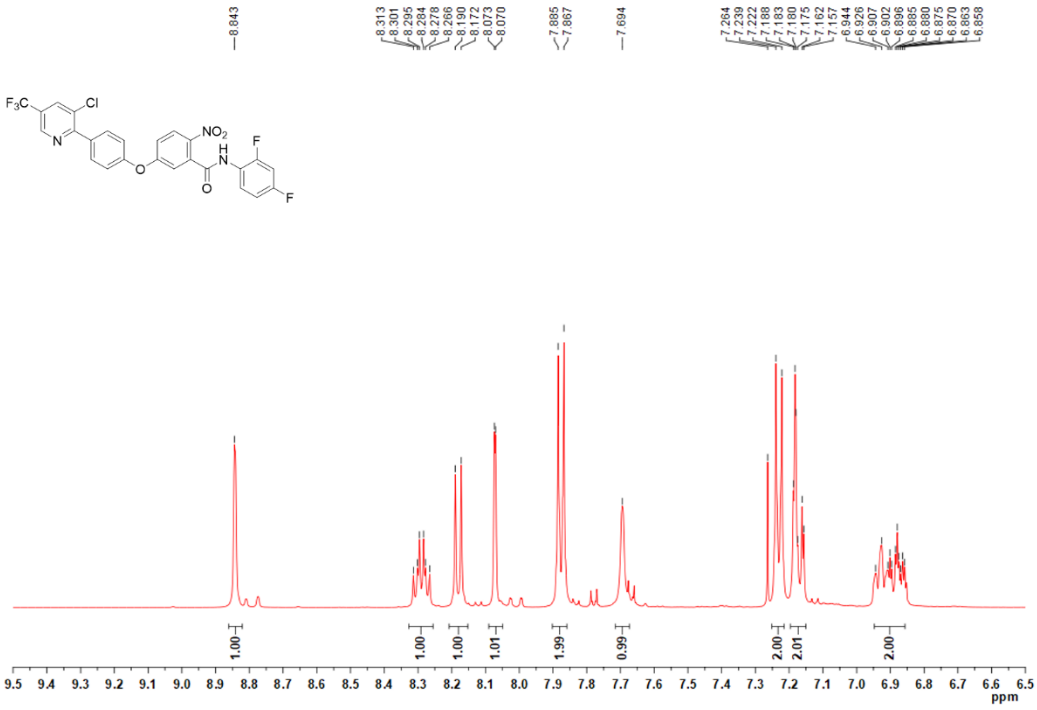

Figure S17. The ^{1}H NMR spectrum of **5i.**

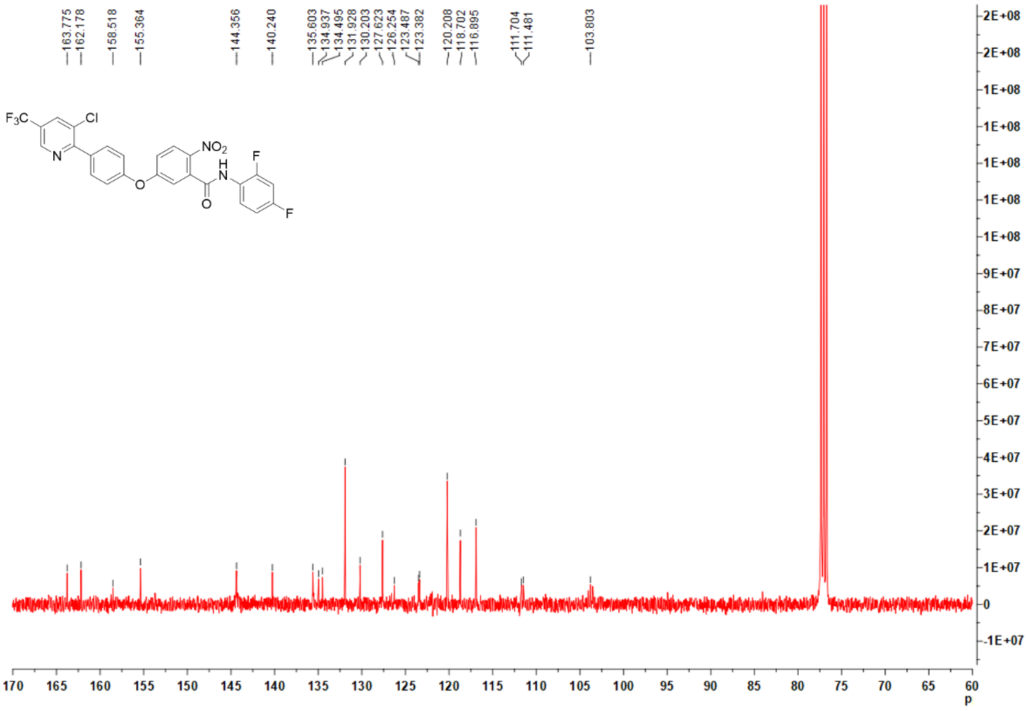

Figure S18. The ^{13}C NMR spectrum of **5i.**

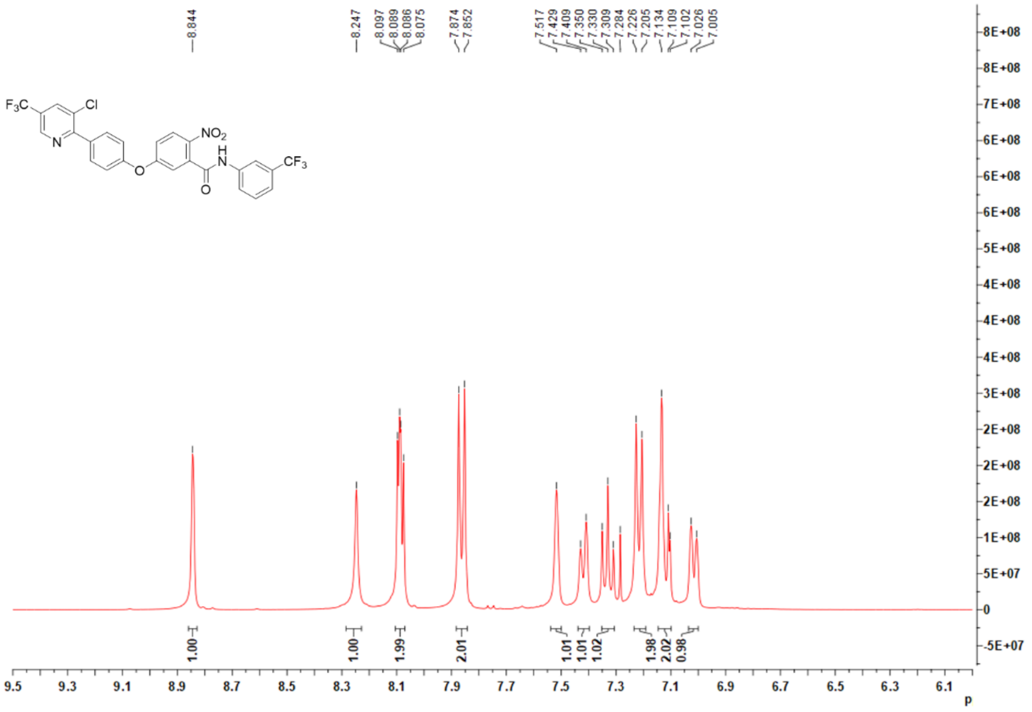

Figure S19. The ^{1}H NMR spectrum of **5j.**

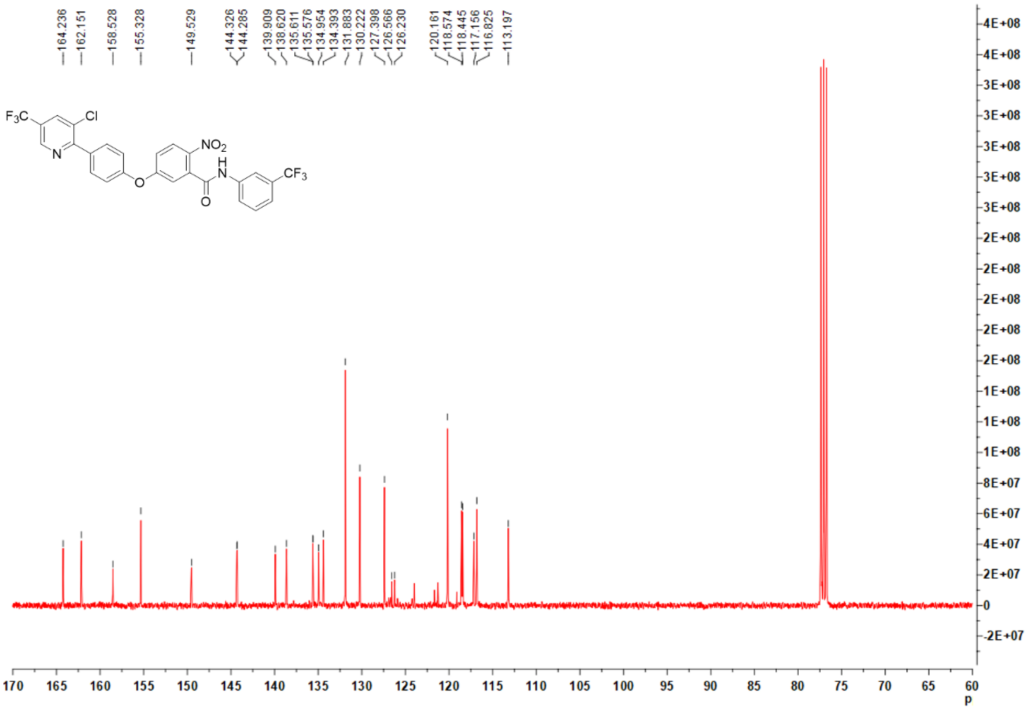

Figure S20. The ^{13}C NMR spectrum of **5j.**

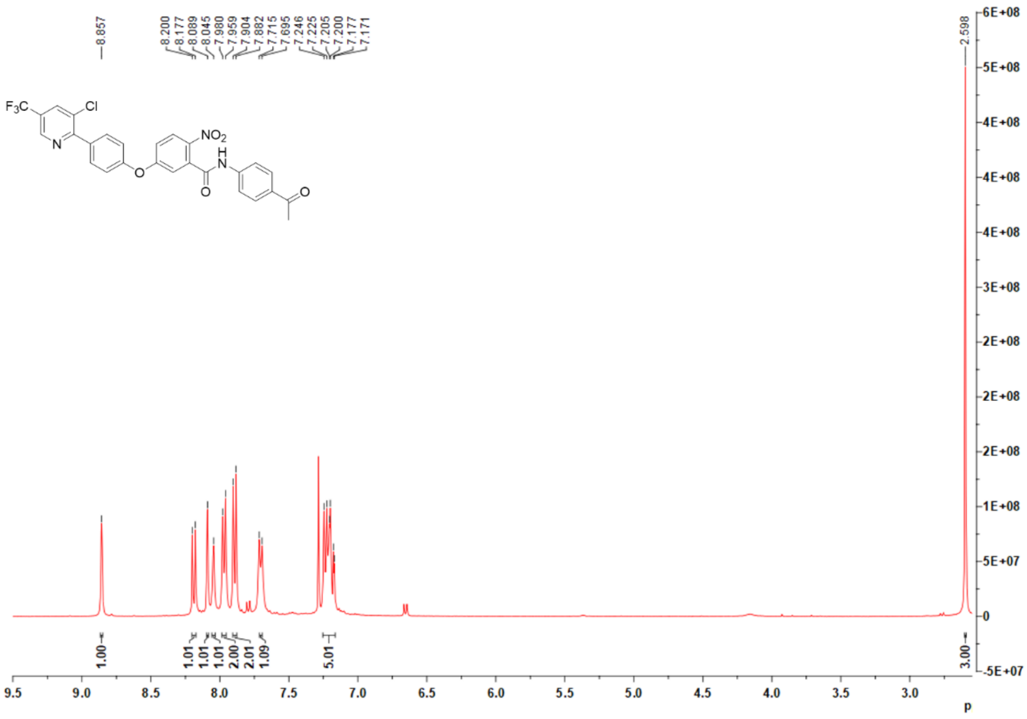

Figure S21. The ^{1}H NMR spectrum of **5k.**

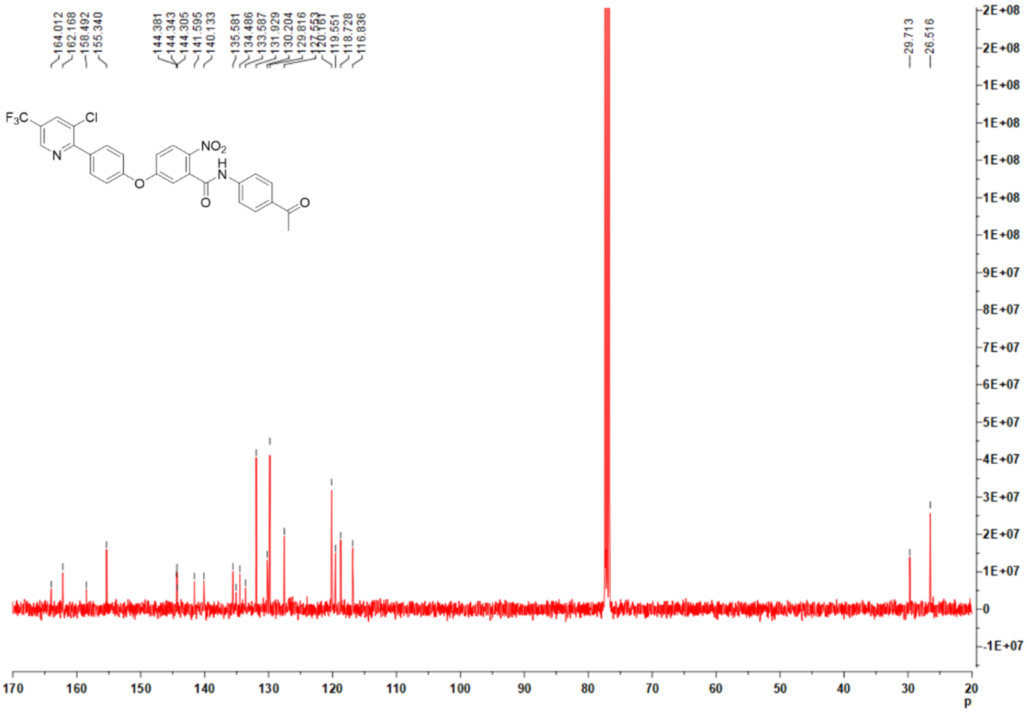

Figure S22. The ^{13}C NMR spectrum of **5k.**
